# Supplementary material for: Development of Ebola virus disease prediction scores: Screening tools for Ebola suspects at the triage-point during an outbreak
Source: PLoS One. 2022 Dec 16;17(12):e0278678. doi: 10.1371/journal.pone.0278678 (PMC9757576; doi:10.1371/journal.pone.0278678)
Supplement: S1 Table — (DOCX) [file pone.0278678.s001.docx]

**S1 Table. Distribution of clinical prediction score (CPS) and extended clinical prediction score (ECPS) among suspect cases**

| **Score** | **Total number of suspects of EVD with the score**  **n (%)** | **Total number of non-EVD with the score**  **n (%)** | **Total number of EVD with the score**  **n (%)** |
| --- | --- | --- | --- |
|  | **N = 10432** | **N = 9871** | **N = 651** |
| **Clinical prediction score (CPS)** |  |  |  |
| **-2** | 7 (0.07) | 7 (0.07) | 0 (0.00) |
| **-1** | 4229 (40.54) | 4134 (42.27) | 95 (14.59) |
| **0** | 5207 (49.91) | 4847 (49.56) | 360 (55.30) |
| **+1** | 632 (6.06) | 550 (5.62) | 82 (12.60) |
| **+2** | 268 (2.57) | 210 (2.15) | 58 (8.91) |
| **+3** | 58 (0.56) | 20 (0.20) | 38 (5.84) |
| **+4** | 17 (0.16) | 6 (0.06) | 11 (1.69) |
| **+5** | 9 (0.09) | 6 (0.06) | 3 (0.46) |
| **+6** | 4 (0.04) | 1 (0.01) | 3 (0.46) |
| **+7** | 1 (0.01) | 0 (0.00) | 1 (0.15) |
| **Extended clinical prediction score (ECPS)** |  |  |  |
| **-4** | 7 (0.07) | 7 (0.07) | 0 (0.00) |
| **-3** | 16 (0.15) | 16 (0.16) | 0 (0.00) |
| **-2** | 3606 (34.57) | 3593 (36.73) | 13 (2.00) |
| **-1** | 4474 (42.89) | 4349 (44.46) | 125 (19.20) |
| **0** | 642 (6.15) | 591 (6.04) | 51 (7.83) |
| **+1** | 662 (6.35) | 560 (5.73) | 102 (15.67) |
| **+2** | 824 (7.90) | 570 (5.83) | 254 (39.02) |
| **+3** | 150 (1.44) | 81 (0.83) | 69 (10.60) |
| **+4** | 26 (0.25) | 8 (0.08) | 18 (2.76) |
| **+5** | 14 (0.13) | 4 (0.04) | 10 (1.54) |
| **+6** | 6 (0.06) | 2 (0.02) | 4 (0.61) |
| **+7** | 3 (0.03) | 0 (0.00) | 3 (0.46) |
| **+8** | 1 (0.01) | 0 (0.00) | 1 (0.15) |
| **+9** | 1 (0.01) | 0 (0.00) | 1 (0.15) |

EVD: Ebola virus disease
