# Supplementary material for: Development of Ebola virus disease prediction scores: Screening tools for Ebola suspects at the triage-point during an outbreak
Source: PLoS One. 2022 Dec 16;17(12):e0278678. doi: 10.1371/journal.pone.0278678 (PMC9757576; doi:10.1371/journal.pone.0278678)
Supplement: S2 Table — (DOCX) [file pone.0278678.s002.docx]

**S2 Table. 10-fold Cross-validation accuracy for clinical prediction score and extended clinical prediction score**

|  | **CPS** | | | **ECPS** | | |
| --- | --- | --- | --- | --- | --- | --- |
|  | **AUCCV** | **Sensitivity (%)** | **Specificity (%)** | **AUCCV** | **Sensitivity %** | **Specificity %** |
| **Resample** |  |  |  |  |  |  |
| **Fold 01** | 0.72 | 99.8 | 9.2 | 0.89 | 98.8 | 16.9 |
| **Fold 02** | 0.74 | 99.9 | 6.2 | 0.84 | 99.0 | 0.2 |
| **Fold 03** | 0.68 | 100.0 | 3.0 | 0.84 | 99.0 | 10.6 |
| **Fold 04** | 0.74 | 99.7 | 4.6 | 0.87 | 99.0 | 18.5 |
| **Fold 05** | 0.72 | 99.6 | 6.2 | 0.91 | 98.9 | 15.4 |
| **Fold 06** | 0.69 | 99.6 | 7.7 | 0.87 | 98.9 | 10.8 |
| **Fold 07** | 0.68 | 99.8 | 6.2 | 0.87 | 98.5 | 0.2 |
| **Fold 08** | 0.73 | 99.9 | 10.8 | 0.90 | 99.0 | 0.2 |
| **Fold 09** | 0.69 | 99.9 | 4.6 | 0.85 | 99.0 | 21.5 |
| **Fold 10** | 0.72 | 99.9 | 6.2 | 0.87 | 98.7 | 15.4 |
| **Mean AUCCV** | **0.71** | **99.8** | **6.5** | **0.87** | **99.0** | **16.9** |

AUCCV: cross-validated area under ROC curve

CPS: clinical prediction score

ECPS: extended clinical prediction score
