# Supplementary material for: Development of Ebola virus disease prediction scores: Screening tools for Ebola suspects at the triage-point during an outbreak
Source: PLoS One. 2022 Dec 16;17(12):e0278678. doi: 10.1371/journal.pone.0278678 (PMC9757576; doi:10.1371/journal.pone.0278678)
Supplement: S1 File — (PDF) [file pone.0278678.s003.pdf]

FICHE DE NOTIFICATION DE FIEVRE HEMORRAGIQUE VIRALE

Numéro d'identification du patient:

Autre numéro d'identification :

Date de Notification: ____/____/_____ (J, M, A)

Section 1. Information sur le Patient

Nom de Famille: ________________________ Autres Noms:_______________________________ Age: _______ ☐ Années ☐ Mois

Sexe: ☐ Masculin ☐ Féminin Numéro de Téléphone (Patient/Famille):_________________ Propriétaire du Téléphone: ______________

Etat du patient au moment de la collecte d'information: ☐ Vivant ☐ Décédé *Si décédé*, Date du Décès: ___/___/____ (J, M, A)

Lieu de Résidence Permanente:

Nom du Chef de Famille: __________________________ Village/Quartier : ______________________Zone de Santé : ________________

Pays de Résidence: _____________________ Province:________________________ Aire de Santé: ________________________

Occupation:

☐ Planteur/Eleveur ☐ Boucher ☐ Chasseur/Vendeur de viande de brousse ☐ Mineur ☐ Chef religieux

☐ Ménagère ☐ Etudiant ☐ Enfant ☐ Médecin traditionnel

☐ Commerçant; type de commerce: ________________________ ☐ Transporteur; type de transport: __________________________

☐ Personnel de santé; position: ________________________ nom du centre médical: _______________________________

☐ Autre; précisez le métier: ____

Endroit où le Patient est Tombé Malade:

Village/Quartier: __________________________ Zone de santé : __________________________ Aire de Santé : __________________

Coordonnées GPS de la maison: Latitude:________________________ Longitude: _______________________

Si ce n'est pas la résidence permanente, Dates de résidence dans cet endroit: ___/__/____ - ___/__/____ (J, M, A)

Section 2. Signes Cliniques et Symptômes

Date de début des signes et symptômes: ____/____/_____ (J, M, A)

Cochez tous les signes et symptômes observés ou ressentis entre la date de début de la maladie et la date de notification:

Signe	Oui	Non	Inc
Fièvre	☐	☐	☐
Si oui, Temp: ____º C Mesure: ☐ Creux Axillaire ☐ Bouche ☐ Rectale			
Nausées / Vomissements	☐	☐	☐
Diarrhées	☐	☐	☐
Fatigue générale intense	☐	☐	☐
Perte d'appétit / anorexie	☐	☐	☐
Douleurs abdominales	☐	☐	☐
Douleurs thoraciques	☐	☐	☐
Douleurs musculaires	☐	☐	☐
Douleurs articulaires	☐	☐	☐
Céphalées	☐	☐	☐
Toux	☐	☐	☐
Difficultés à respirer	☐	☐	☐
Difficultés à avaler	☐	☐	☐
Mal à la gorge	☐	☐	☐
Ictère (conjonctives/gencives/peau)	☐	☐	☐
Conjonctivite (œil rouge)	☐	☐	☐
Eruptions cutanées	☐	☐	☐
Hoquet	☐	☐	☐
Douleurs retro-orbitaires/photophobie	☐	☐	☐
Coma / perte de conscience	☐	☐	☐
Confusion ou désorientation	☐	☐	☐

**Saignements**	Oui	Non	Inc
Si Oui:			
Saignements des gencives	☐	☐	☐
Saignements aux sites d'injections	☐	☐	☐
Saignements du nez (épistaxis)	☐	☐	☐
Selles rouges ou noires (mélénas)	☐	☐	☐
Vomissements sanglants (hématémèses)	☐	☐	☐
Vomissement noirâtre (vomito negro)	☐	☐	☐
Toux sanglante (hémoptysie)	☐	☐	☐
Saignements vaginaux, en dehors des règles	☐	☐	☐
Hématomes / Pétéchies / purpura	☐	☐	☐
Sang dans les urines (hématurie)	☐	☐	☐
Autres signes hémorragiques	☐	☐	☐

Si oui, précisez: ____________________________

Autres signes cliniques non-hémorragiques: ☐ Oui ☐ Non ☐ Inc

Si oui, précisez: ___________________________

Section 3. Informations sur l'hospitalisation

Au moment de cette notification, le malade est-il déjà hospitalisé ou en cours d'admission à l'hôpital? ☐ Oui ☐ Non

Si oui, Date d'hospitalisation: ____/____/_____ (J, M, A) Nom de l'hôpital: ____

Village/Ville: ________________________ Zone de Santé: ______________________ Aire de Santé: ___________________

Le malade est-il en isolement/en cours d'isolement? ☐ Oui ☐ Non *Si oui, D*ate d'isolement: ____/____/_____ (J, M, A)

Le malade était-il hospitalisé ailleurs ou a visité un centre de soins pour la maladie actuelle? ☐ Oui ☐ Non ☐ Inc

Si oui, veuillez compléter une ligne ci-dessous pour chacune des hospitalisations précédentes:

Dates d'hospitalisation	Nom du centre en médical	Village	Zone de santé	Le patient était-il isolement?
___/__/____ - ___/__/____ (J, M, A)				☐ Oui ☐ Non
___/__/____ - ___/__/____ (J, M, A)				☐ Oui ☐ Non

Numéro d'identification du patient:

Section 4. Epidémiologie / Facteurs d'expositions

PENDANT LE MOIS PRÉCÉDENT LE DÉBUT DES SYMPTÔMES:

1. Il y a-t-il eu contacts avec un malade Ebola, connu/suspect, ou simplement avec une personne malade? ☐ Oui ☐ Non ☐ Inc

Si oui, veuillez compléter une ligne ci-dessous pour chacun des malades pouvant être une source de contamination:

Nom du malade potentiel	Lien de parenté	Date(s) du contact (J, M, A)	Village	Préfecture	Est-ce-que la personne était vivante ou décédée ?	Types de contact**
		___/___/___ - ___/___/___			☐ Vivante<br>Décédée, Date: ___/___/___ (J, M, A)	
		___/___/___ - ___/___/___			☐ Vivante<br>Décédée, Date: ___/___/___ (J, M, A)	
		___/___/___ - ___/___/___			☐ Vivante<br>Décédée, Date: ___/___/___ (J, M, A)	

****Type de contact: (indiquez toutes les possibilités)**

1 – A touché des secrétions/excrétions du malade (sang, vomissures, salive, urine, selles)
2 – A touché directement le corps du malade (vivant ou décédé)
3 – A touché ou partagé linges, habits, plats/assiettes, instruments avec le malade
4 – A dormi ou mangé avec, ou séjourné dans la même maison ou pièce que le malade

2. Est-ce-que le patient a participé à des funérailles <u>avant</u> la maladie actuelle? ☐ Oui ☐ Non ☐ Inc

Si oui, veuillez compléter une ligne ci-dessous pour chacune des participations à un enterrement:

Nom de la personne décédée	Lien de parenté	Dates de participation aux funérailles (J, M, A)	Village	Préfecture	Avez-vous porté ou touché le corps?
		___/___/___ - ___/___/___			☐ Oui ☐ Non
		___/___/___ - ___/___/___			☐ Oui ☐ Non

3. Le patient a-t-il voyagé en dehors de chez lui ou de son village/ville <u>avant</u> la maladie actuelle? ☐ Oui ☐ Non ☐ Inc

Si oui, Village: ________________________ Zone de santé : __________________ Date(s): ___/___/____ - ___/___/____ (J, M, A)

4. Le patient a-t-il été hospitalisé, a-t-il consulté dans un hôpital ou visité quelqu'un hospitalisé <u>avant</u> la maladie actuelle? ☐Oui ☐Non ☐Inc

Si oui, Nom du patient: ____________________ Date(s): ___/___/____ - ___/___/____ (J, M, A)

Nom du Centre Médical: ________________________ Village: ______________________ Zone de Santé: ________________

5. Le patient a-t-il consulté un médecin traditionnel <u>avant</u> la maladie actuelle? ☐ Oui ☐ Non ☐ Inc

Si oui, Nom du Médecin: ________________Village: _______________ Zone de santé: _____________ Date: ___/___/____ (J, M, A)

6. Le patient a-t-il eu un contact direct (chasse, touché, mangé) avec des animaux ou de la viande crue <u>avant</u> de tomber malade? ☐Oui ☐Non ☐Inc

Si oui, cochez les cases nécessaires:

Animal:	Status (check one only):
☐ Chauve-souris (ou excrétions de)	☐ En bonne santé ☐ Malade/Mort
☐ Singes	☐ En bonne santé ☐ Malade/Mort
☐ Rongeurs (ou excrétions de)	☐ En bonne santé ☐ Malade/Mort
☐ Cochons	☐ En bonne santé ☐ Malade/Mort
☐ Volaille ou oiseaux sauvages	☐ En bonne santé ☐ Malade/Mort
☐ Vaches, chèvres, ou moutons	☐ En bonne santé ☐ Malade/Mort
☐ Autres; *précisez :*_____________	☐ En bonne santé ☐ Malade/Mort

7. Est-ce-que le patient a eu une piqûre de tique dans les 2 dernières semaines? ☐ Oui ☐ Non ☐ Inc

Section 5. Prélèvements Biologiques pour le Laboratoire

<u>Mode de prélèvements et d'envoi :</u>

- Identifier le tube: **nom**, **date de prélèvement** et le **numéro d'identification du malade**
- Envoyer les échantillons avec **réfrigération,** and **emballés correctement**.
- Prélever le sang complet dans un tube EDTA (bouchon violet) tube – si non disponible, bouchon vert (héparine) ou rouge (sans anticoagulant) sont acceptables
- **Volume demandé = <u>4ml</u>** (volume minimum = 2ml)

Est-ce qu'un prélèvement a déjà été soumis pour ce malade? ☐ Oui ☐ Non

<u>Prélèvement 1:</u> *Ne pas remplir*

Date du prélèvement: ____/____/______ (J, M, A)

Type de prélèvement:

☐ Sang complet
☐ Ponction cardiaque (*post-mortem)*
☐ Biopsie de peau
☐ Autre prélèvement, précisez: ________________

<u>Prélèvement 2:</u> *Ne pas remplir*

Date du prélèvement: ____/____/______ (J, M, A)

Type de prélèvement:

☐ Sang complet
☐ Ponction cardiaque (*post-mortem)*
☐ Biopsie de peau
☐ Autre prélèvement, précisez: ________________

Section 6. Fiche de notification complétée par:

Nom: ______________________________ Téléphone: _________________________ E-mail: ____________________________Role:

<u>ZS</u> : _________________________ Centre médical: ____

Informations fournies par ☐ Patient ☐ Représentant; *Si représentant*, Nom:______________________ Lien de parenté: ______________

Nom du Patient: ______

Numéro Identification du Patient: ______

***Si le patient est décédé ou est déjà convalescent ou guéri, veuillez remplir la section suivante.**
***Si le malade va être admis à l'hôpital, ne complétez pas la section suivante, (ce sera fait lors de la sortie)**

Section 7. Statut final du patient

Veuillez remplir cette section lorsque le patient est guéri et sort de l'hôpital ou lors de son décès.

Date à laquelle les informations sont rapportées: ____/____/_____ (J, M, A)

Statut final du patient: ☐ Vivant ☐ Décédé

Est-ce-que le patient a eu des signes hémorragiques inexpliqués pendant la durée de la maladie? ☐ Oui ☐ Non ☐ Inc

Si oui, veuillez préciser: ______________________________

Si le malade est guéri et sort de l 'hôpital :

Nom de l'hôpital: ______________________ Zone de Santé: ______________________

Si le malade était en isolement, date de sortie de la zone d'isolement: ____/____/______ (J, M, A)

Date de sortie de l'hôpital: ____/____/______ (J, M, A)

Si le malade est décédé:

Date du décès: ____/____/______ (J, M, A)

Lieu du décès: ☐ Domicile ☐ Hôpital: ______________ ☐ Ailleurs: ______________

Village : ______________ Zone de santé: ______________ Aire de santé: ______________

Date des funérailles: ____/____/______ (J, M, A) Funérailles organisées par: ☐ Famille/communauté ☐ Equipe d'enterrement

Lieu des funérailles/enterrement:

Village: ______________ Zone de Santé: ______________ Aire de Santé: ______________

Veuillez cocher une réponse pour tous les signes et symptômes, indiquant s'ils ont été trouvés ou non pendant toute la durée de la maladie (en tenant compte également de la période d'hospitalisation):

Fièvre	☐ Oui	☐ Non	☐ Inc
Si oui, Temp: ____º C Mesure: ☐ Creux Axillaire ☐ Bouche ☐ Rectale			
Nausées / Vomissements	Oui	Non	Inc
Diarrhées	☐ Oui	☐ Non	☐ Inc
Fatigue générale intense	Oui	Non	Inc
Perte d'appétit / Anorexie	☐ Oui	☐ Non	☐ Inc
Douleurs abdominales	Oui	Non	Inc
Douleurs thoraciques	☐ Oui	☐ Non	☐ Inc
Douleurs musculaires	Oui	Non	Inc
Douleurs articulaires	☐ Oui	☐ Non	☐ Inc
Céphalées	Oui	Non	Inc
Toux	☐ Oui	☐ Non	☐ Inc
Difficultés à respirer	Oui	Non	Inc
Difficultés à avaler	☐ Oui	☐ Non	☐ Inc
Mal à la gorge	Oui	Non	Inc
Ictère (conjonctives/gencives/peau)	☐ Oui	☐ Non	☐ Inc
Conjonctivite (œil rouge)	Oui	Non	Inc
Eruptions cutanées	☐ Oui	☐ Non	☐ Inc
Hoquet	Oui	Non	Inc
Douleurs retro-orbitaires/photophobie	☐ Oui	☐ Non	☐ Inc
Coma / perte de conscience	Oui	Non	Inc
Confusion ou désorientation	☐ Oui	☐ Non	☐ Inc

Autres signes ou symptômes cliniques non hémorragiques: ☐ Oui ☐ Non ☐ Inc

Si oui, Veuillez précisez: ____________________________
