# Supplementary material for: Development of Ebola virus disease prediction scores: Screening tools for Ebola suspects at the triage-point during an outbreak
Source: PLoS One. 2022 Dec 16;17(12):e0278678. doi: 10.1371/journal.pone.0278678 (PMC9757576; doi:10.1371/journal.pone.0278678)
Supplement: S2 File — (DOCX) [file pone.0278678.s004.docx]

| **Section/Topic** | **Item** |  | **Checklist Item** | **Page** | **Text extracts** |
| --- | --- | --- | --- | --- | --- |
| **Title and abstract** | | | | |  |
| Title | 1 | D;V | Identify the study as developing and/or validating a multivariable prediction model, the target population, and the outcome to be predicted. | 1 | Development of Ebola Virus Disease prediction scores: screening tools for Ebola suspects at the triage-point during an outbreak |
| Abstract | 2 | D;V | Provide a summary of objectives, study design, setting, participants, sample size, predictors, outcome, statistical analysis, results, and conclusions. | 2−3 | Background: The control of Ebola virus disease (EVD) outbreaks relies on rapid diagnosis and prompt action, a daunting task in limited-resource contexts.  This study develops prediction scores that can help healthcare workers improve their decision-making at the triage-point of EVD suspect-cases during EVD outbreaks.  Methods: We computed accuracy measurements of EVD predictors to assess their diagnosing ability compared to the reference standard GeneXpert® results, during the eastern DRC EVD outbreak. We developed predictive scores using the Spiegelhalter-Knill-Jones approach and constructed a clinical prediction score (CPS) and an extended clinical prediction score (ECPS). We plotted the receiver operating characteristic curves (ROCs), estimated the area under the ROC (AUROC) to assess the performance of scores, and computed net benefits (NB) to assess the clinical utility (decision-making ability) of the scores at a given cut-off. We performed decision curve analysis (DCA) to compare, at a range of threshold probabilities, prediction scores’ decision-making ability and to quantify the number of unnecessary isolation.  Results: The analysis was done on data from 10432 subjects, including 651 EVD cases. Fatigue, difficulty swallowing, red eyes, gingival bleeding, hematemesis, confusion, hemoptysis, and history of contact with an EVD case were predictors of EVD. The AUROC for ECPS was 0.88 (95%CI: 0.86–0.89), significantly greater than this for CPS, 0.71 (95%CI: 0.69–0.73) (p < 0.0001).  At -1 point of score, the CPS yielded a sensitivity of 85.4% and specificity of 42.3%, and the ECPS yielded a sensitivity of 78.8% and specificity of 81.4%. At 10% of threshold probability, ECPS gave an NB of 0.033 and a net reduction of unnecessary isolation of 67.1%. Using ECPS as a joint approach to isolate EVD suspects reduces the number of unnecessary isolation by 65.7%.  Conclusion: The scores developed in our study showed a good performance as EVD case predictors since their use improved the net benefit, i.e., their clinical utility. These rapid and low-cost tools can help in decision-making to isolate EVD suspect-cases at the triage-point during an outbreak. However, these tools still require external validation and cost-effectiveness evaluation before any use at a large scale. |
| **Introduction** | | | | |  |
| Background and objectives | 3a | D;V | Explain the medical context (including whether diagnostic or prognostic) and rationale for developing or validating the multivariable prediction model, including references to existing models. | 3−5 | The control of EVD outbreaks relies on accurate and early detection of the pathogen to allow the rapid implementation of countermeasures [9]. Since the 2018 EVD outbreak [Equateur Province, Democratic Republic of the Congo (DRC)], the Cepheid® GeneXpert Ebola platform was used following the WHO’s instructions as the reference standard for EVD diagnosis at the point of care. The GeneXpert® is a highly sensitive and specific, rapid, and fully automated dual-purpose quantitative RT-PCR that diagnoses EVD with a few skills and a short turn-around time [10]. Although on-site GeneXpert technology has shown beneficial results in controlling the EVD outbreaks [11], timely scaling-up of this technology face to an expanding or quickly changing outbreak context remains challenging because of the requirements such as electricity supply, laboratory infrastructure, personnel training, and the cost. In these contexts, and an absence of validated rapid diagnostic tests (RDTs), clinical signs and symptoms associated with epidemiological information can serve as an immediate tool to identify suspect-cases and to support patients’ categorization and management. Early and accurate recognition of EVD can help improve the healthcare workers' (HCW) decision-making and epidemic control.  In the early stage of the infection, sudden fever onset and other non-specific symptoms such as severe headache, muscle and joint pain, and fatigue characterize the EVD [12]. In tropical settings, many infectious diseases have a similar initial clinical presentation. EBOV-specific symptoms appear at the last stage further delaying the diagnostic in absence of detection tools.  Often, HCWs must decide on isolating suspected cases without having any timely access to diagnostic tests. HCWs must weigh up the risk of sending true-positive EVD cases back to the community against the risk of admitting false-positive cases into the isolation wards. These errors in the triage result in the spread of the infection into the community or sustain nosocomial transmission in health settings, both delaying timely access to appropriate EVD care.  As for other epidemic pathologies, the clinical case definitions were developed, combining the signs and symptoms to risk factors (as having had contact with a confirmed case). The WHO clinical case definition (or local adaptation) is most commonly used through a recent meta-analysis showed its sub-optimal performance (pooled sensitivity of 81.5% (95% CI 74.1–87.2), and specificity 35.7% (28.5–43.6)) [13, 14].  Thus, further research on simple clinical scoring systems is needed to add precision to the risk assessment in a single patient. Such simple tools, as for other conditions, are used, to better classify patients either for prognostic or management purposes [15–18]. |
|  | 3b | D;V | Specify the objectives, including whether the study describes the development or validation of the model or both. | 6 | This study aims 1) to develop an EVD clinical prediction rule using surveillance data from the 2018–2020 DRC EVD outbreak; 2) to evaluate the clinical usefulness of the prediction rule to improve healthcare workers’ decision-making at the triage-point of EVD suspect-cased during outbreaks. |
| **Methods** | | | | |  |
| Source of data | 4a | D;V | Describe the study design or source of data (e.g., randomized trial, cohort, or registry data), separately for the development and validation data sets, if applicable. | 6 | In this retrospective cohort study, we analyzed the epidemiological and clinical data collected routinely upon identification or admission of an EVD suspect case. We used the datasets of the surveillance and care units teams deployed in the areas covered by the EVD coordination team of Butembo, in the North-Kivu province. |
|  | 4b | D;V | Specify the key study dates, including start of accrual; end of accrual; and, if applicable, end of follow-up. | 6 | We included data with alive EVD suspects and the community deaths identified in the Butembo area between September 3, 2018 and February 17, 2020. |
| Participants | 5a | D;V | Specify key elements of the study setting (e.g., primary care, secondary care, general population) including number and location of centres. | 6−7 | We used the datasets of the surveillance and care units teams deployed in the areas covered by the EVD coordination team of Butembo, in the North-Kivu province. We included data with alive EVD suspects and the community deaths identified in the Butembo area between September 3, 2018 and February 17, 2020.  In this outbreak, the surveillance and clinical teams used a standard clinical case definition adapted from the WHO Integrated Disease Surveillance and Response (IDSR) guidelines to identify EVD suspect-cases [23]. |
|  | 5b | D;V | Describe eligibility criteria for participants. | 7 | In this outbreak, the surveillance and clinical teams used a standard clinical case definition adapted from the WHO Integrated Disease Surveillance and Response (IDSR) guidelines to identify EVD suspect-cases [23].  The case definition contained notions of residence, signs, or symptoms without referring to contact information. The case definition was stated as follows: “*Any person alive or dead, living in North-Kivu, South-Kivu, or Ituri provinces or any person who traveled to these provinces during this period and who reported the followings signs or symptoms; sudden fever onset and at least three of the following symptoms: vomiting, diarrhea, abdominal pain, conjunctivitis, rash, unexplained bleeding from any part of the body, muscle pain, intense fatigue, difficulty of swallowing, the difficulty of breathing, hiccups, or headache*.”  . |
|  | 5c | D;V | Give details of treatments received, if relevant. | 7 | After the clinical and epidemiological assessment, each EVD suspect-case was submitted to the sample collection process (blood or oral swab) for confirmatory testing. While waiting for the result, EVD suspect patients were kept in the isolation wards within the triage center (TC), Ebola treatment center (ETC), or in the community for cases associated with refusal to be transferred at the care units.  For the Butembo areas, all samples from suspect-cases were tested in the EVD field laboratory setup by the Institut National de Recherche Biomédicale (INRB) in Butembo. The confirmation of EBOV infection relied on the detection EBOV Nucleoprotein (NP) and/or Glycoprotein (GP) in the clinical specimens through GeneXpert® (Cepheid, Sunnyvale, CA, USA) [10,11]. |
| Outcome | 6a | D;V | Clearly define the outcome that is predicted by the prediction model, including how and when assessed. | 7-8 | In our analysis, we used the GeneXpert® results as the reference for the EVD status. EVD confirmed case was defined as a suspect case with a GeneXpert® positive, i.e., EBOV Cycle threshold-values (Ct-values) NP < 40. A probable EVD case was defined as a suspect case with high-risk contact for whom a test was not available, because no sample was collected. A non-case was a suspect case with a GeneXpert® negative result. |
|  | 6b | D;V | Report any actions to blind assessment of the outcome to be predicted. | _ | NA |
| Predictors | 7a | D;V | Clearly define all predictors used in developing or validating the multivariable prediction model, including how and when they were measured. | 7 | The case definition contained notions of residence, signs, or symptoms without referring to contact information. The case definition was stated as follows: “Any person alive or dead, living in North-Kivu, South-Kivu, or Ituri provinces or any person who traveled to these provinces during this period and who reported the followings signs or symptoms; sudden fever onset and at least three of the following symptoms: vomiting, diarrhea, abdominal pain, conjunctivitis, rash, unexplained bleeding from any part of the body, muscle pain, intense fatigue, difficulty of swallowing, the difficulty of breathing, hiccups, or headache.”  For each EVD suspect case, the surveillance and/or clinical team collected, guided by a standard notification form, data on socio-demographics (age, gender, residence, profession), putative epidemiologic routes of contagion (contacts, type of contact), date of symptoms onset, and signs and symptoms at the time of diagnostic. |
|  | 7b | D;V | Report any actions to blind assessment of predictors for the outcome and other predictors. | _ | NA |
| Sample size | 8 | D;V | Explain how the study size was arrived at. | ­_ | **NA** |
| Missing data | 9 | D;V | Describe how missing data were handled (e.g., complete-case analysis, single imputation, multiple imputation) with details of any imputation method. | 9 | We selected the adjusted diagnostic predictors with aLR ≥1.5 or ≤ 0.67 to construct the scoring system. We obtained the diagnostic score for each retained predictor from the natural log-transformation of the aLRs, rounded to their nearest integer. Positive scores favored the presence of EVD whereas negative scores argued against it. We obtained the total predictive score (X) for each suspect by summing the diagnostic score for each predictor. We assigned a value of 0 to missing data. |
| Statistical analysis methods | 10a | D | Describe how predictors were handled in the analyses. | 8 | We dichotomized continuous measurements at the cut-off where the sum of specificity and sensitivity was maximized using the receiver operating characteristic curves (ROC). |
|  | 10b | D | Specify type of model, all model-building procedures (including any predictor selection), and method for internal validation. | 9−10 | We selected associated predictors with a crude LR+ ≥2.0 or LR - ≤0.5 and adjusted in multivariate logistic regression analysis according to an adaptation of the Spiegelhalter-Knill-Jones approach [24] as described in [25]. This approach combines the independent Bayes method and the logistic regression to construct a scoring system [26]. We selected the adjusted diagnostic predictors with aLR ≥1.5 or ≤ 0.67 to construct the scoring system. We obtained the diagnostic score for each retained predictor from the natural log-transformation of the aLRs, rounded to their nearest integer. |
|  | 10c | V | For validation, describe how the predictions were calculated. | 10 | We selected the adjusted diagnostic predictors with aLR ≥1.5 or ≤ 0.67 to construct the scoring system. We obtained the diagnostic score for each retained predictor from the natural log-transformation of the aLRs, rounded to their nearest integer. Positive scores favored the presence of EVD whereas negative scores argued against it. We obtained the total predictive score (X) for each suspect by summing the diagnostic score for each predictor. We assigned a value of 0 to missing data. Finally, we computed from the total score for each suspect, the posterior probability of having EVD as follows:  $P\left( D \vert\boldsymbol{X} \right)=\frac{\exp\left( \beta0+\sum\beta i*Xi \right)}{1+\exp\left( \beta0+\sum\beta i*Xi \right)}$(1)  Where X is the vector of all predictors Xi, D the EVD status, β0 is the constant of the regression equation and βi the coefficient of the Xi predictor. |
|  | 10d | D;V | Specify all measures used to assess model performance and, if relevant, to compare multiple models. | 10−13 | Thus, we evaluated the discrimination performance of the CPS and ECPS by constructing their receiver operating characteristic curves (ROC) and calculating the areas under the curve (AUROC) with 95% confidence intervals (95%CIs) in the whole dataset and in subgroups of the EVD suspect’s dataset (e.g., fitting or not the WHO case definition). An area of 1.0 indicates perfect discrimination power, whereas an area of 0.5 indicates no discrimination of the binary disease status (EVD versus non-EVD). Further, we computed the diagnostic accuracy measures, e.g. sensitivity, specificity, PPV, and NPV, and their binomial 95%CIs at each cut-off point value of the score for each prediction model. Finally, we compared the AUROCs for prediction models developed by computing the critical ratio z as described in Hanley and McNeil [28]. To evaluate the over-fitting of our models, we plotted 10-fold calibration plots of the models. Calibration refers to the agreement between predicted probabilities and observed proportions, or the actual risk of a clinical outcome. We used the Hosmer-Lemeshow goodness-of-fit test, and a model was considered statistically fit data if the p-value was greater than 0.1.  To evaluate the clinical usefulness or utility of the developed prediction scores, we performed decision curve analysis, which depicts the net benefit (NB) of using a model at various ranges of threshold probabilities of interest as described by Vickers and Elkin [29].  Additionally, we plotted the decision curves for models and computed the reduction in the number of unnecessary isolation of suspects by using these models at a range of threshold probabilities of interest to compare our developed models. Additionally, the models were compared to two extreme strategies, isolating all suspects (where TP/N is the prevalence of EVD among the suspects and FP/N is one minus the prevalence of EVD in the NB formula) and isolating none of the suspects (where TP = zero and FP = zero, thus NB = zero at any threshold probability).  We computed the reduction in the number of unnecessary isolation per 100 EVD suspect as described in [30]. |
|  | 10e | V | Describe any model updating (e.g., recalibration) arising from the validation, if done. | _ | NA |
| Risk groups | 11 | D;V | Provide details on how risk groups were created, if done. | 9 | The observed probability of Ebola virus disease by prediction score was then calculated. (see item 6c |
| Development vs. validation | 12 | V | For validation, identify any differences from the development data in setting, eligibility criteria, outcome, and predictors. | 6−7 | See Items 4, 5, 6 and 7 above |
| **Results** | | | | |  |
| Participants | 13a | D;V | Describe the flow of participants through the study, including the number of participants with and without the outcome and, if applicable, a summary of the follow-up time. A diagram may be helpful. | 9−10 | **Fig 1. Flow diagram showing the number of EVD suspects in the study and their outcomes.** |
|  | 13b | D;V | Describe the characteristics of the participants (basic demographics, clinical features, available predictors), including the number of participants with missing data for predictors and outcome. | 14−15 | **Table 1. Epidemiological and clinical characteristics of the included patients** |
|  | 13c | V | For validation, show a comparison with the development data of the distribution of important variables (demographics, predictors and outcome). | 14−15 | **Table 1. Epidemiological and clinical characteristics of the included patients** |
| Model development | 14a | D | Specify the number of participants and outcome events in each analysis. | 14−15 | **Table 1. Epidemiological and clinical characteristics of the included patients** |
|  | 14b | D | If done, report the unadjusted association between each candidate predictor and outcome. | 14−17 | **Table 1. Epidemiological and clinical characteristics of the included patients**  **Table 2. Accuracy of demographic, epidemiologic and clinical predictors for the diagnosis of EVD (compared to the reference standard laboratory confirmation)** |
| Model specification | 15a | D | Present the full prediction model to allow predictions for individuals (i.e., all regression coefficients, and model intercept or baseline survival at a given time point). | 18−19 | Note: This is the Spiegelhalter and Knill-Jones method. See adjusted likelihood ratios.  **Table 3. Crude and Weighted score developed for clinical and extended clinical model score rules** |
|  | 15b | D | Explain how to the use the prediction model. | 19−20 | **Fig 2. Barplots depicting the distribution of the Clinical prediction score and Extended Clinical Prediction Score in EVD and Non-EVD groups**  To evaluate the clinical usefulness or utility of the developed prediction scores, we performed decision curve analysis, which depicts the net benefit (NB) of using a model at various ranges of threshold probabilities of interest as described by Vickers and Elkin [29]. The net benefit defines the difference between the benefit for true cases (TP) who would receive correctly the intervention (isolation and care) based on the prediction of the model and the expected harm for non-cases (FP) who will erroneously receive the intervention weighted by the odds of the patient’s threshold probability. The model with the higher net benefit at a given threshold probability is the preferred model  Finally, we evaluated our prediction models according to two additional clinical practice approaches in healthcare settings: joint and conditional tests or approaches. In both approaches, the suspects with no reported risk of exposure would be considered to not have the disease and the clinical team would act accordingly. No additional action, e.g., isolation, would be required. In the joint approach, the clinical team should clinically examine all suspects at low-, intermediate-, and high-risk reported exposure and recommend for isolation only those with a predicted probability of EVD greater than 5% (the cut-off chosen to maximize sensitivity, about 90 percent, in disease adverse context). In the conditional approach, the clinical team should isolate all suspects with high-risk reported exposure irrespective of their predicted probability of the disease and then suspects at low and intermediate reported exposure having an EVD predicted probability greater than 5%. |
| Model performance | 16 | D;V | Report performance measures (with CIs) for the prediction model. | 19−26 | **Fig 3. Receiver operating curves (ROCs) plotting the discriminatory performance of the clinical prediction and extended clinical prediction scores for the screening of EVD**  The mean cross-validation ROC (AUCCV) over the cross-validated samples with the ECPS was 0.87 (0.88 for the full dataset), and the mean AUCCV over the cross-validated samples with CPS was 0.71, the same as for the full dataset**.**  **Fig 4. Calibration plots for clinical prediction (CPS) and extended clinical prediction (ECPS) scores for the screening of EVD.**  **Fig 5. Receiver operating characteristic (ROC) curves for the 10-fold cross validation of both Ebola clinical prediction scores on the test set.**  **Fig 6. Decision curve plotting net benefit of the prediction scores at a range of threshold probability.**  **Table 5. Net benefit at some threshold probabilities and number of avoided unnecessary suspects isolation and /or further testing using the two developed models compared to isolating all suspects** |
| Model-updating | 17 | V | If done, report the results from any model updating (i.e., model specification, model performance). | _ | NA |
| **Discussion** | | | | |  |
| Limitations | 18 | D;V | Discuss any limitations of the study (such as nonrepresentative sample, few events per predictor, missing data). | 26−27 | The main limitations of the study are  First, the database used for this analysis contained many community cases from surveillance, which had missed clinical and epidemiologic data.  Second, the study included a population with a high prevalence of severe patients from the Ebola treatment unit during the epidemic period. Thus, the study missed most clinically asymptomatic or mild cases. Although the performance of our scores was excellent, their use during the inter-epidemic period or in other settings will need further evaluation.  Finally, the DCAs are simple and quick tools to improve the making of decisions, as their construction requires only one parameter, the range of threshold probabilities. However, for more complex decision choices, i.e., to inform policy, further research must capture the uncertainty involved before their use in a large context. |
| Interpretation | 19a | V | For validation, discuss the results with reference to performance in the development data, and any other validation data. | _ | NA |
|  | 19b | D;V | Give an overall interpretation of the results, considering objectives, limitations, results from similar studies, and other relevant evidence. | 26−32 | This study developed two prediction scores, which included fatigue, difficulty in swallowing, confusion-disorientation, red-eyes, gingival bleeding, hematemesis, hemoptysis, and any history of contact with an EVD patient as a diagnostic predictor, some of which and their accuracy performance was consistent with those previously developed [21,22,36]. Although the methods used to develop prediction scores were different from ours, both models demonstrated better performance in discriminating EVD cases from others. Additionally, this discriminative performance remained the same, thus robust, in the two stratified sets of sample, e.g. for the set who fitted or did not fit the WHO case definition for the EVD suspects. The highest sum sensitivity-specificity is at -1 point and -1 point, respectively, for the clinical prediction score and the extended one; the models are associated with sensitivity of 85% and 42.3% and specificity of 78.8% and 81.4%. This means, for our prediction scores, the presence of a positive numeric score will be a useful argument for more investigations and/or the isolation of a suspect case. However, instead of choosing a sole cut-point, it is worth to divide it into three or four categories based on the numerical score, describing different probability levels of being useful in clinical practice. For instance, for these two models, one can consider a score of prediction equal or less than -2 as having a very low probability of the disease (i.e., less than 20%) and a score of prediction greater than +2 as having very high probability of the disease and act accordingly. With intermediate probabilities, e.g., between these two levels of predictive disease probabilities, one should consider the prediction score as not conclusive and call for additional clinical evaluation. In this sense, using these models can allow the decision to be made on at least 40.7 percent (4141 non-EVD and 114 EVD cases) and 79.8% (7965 non-EVD and 360 EVD cases), respectively, for the clinical prediction score and the extended score.  The AUROC is a summary measure of discrimination between individuals who experienced the disease and those who did not. Though it is subjective to determine the good value of the AUROC for assessing the disease risk, the AUROC values for the two scores developed are considered excellent, according to D’Agostino’s AUROC classification [37]. In addition, the two final scoring algorithms are quite robust for the selected variables. However, accuracy measures only tell us about the models or tests' discriminative performance; they do not consider the clinical consequences of using these clinical prediction models to inform on their clinical usefulness in making decisions [29]. While addressing this issue, the study has also shown that incorporating prediction models into the decision-making to isolate suspect-cases would improve clinical results or outcomes linked to the use of WHO case definition for suspect-cases while awaiting the confirmation results. Their use avoids many unnecessary isolations and only few true EVD cases could miss the isolation. Our findings demonstrate the utility of these models while identifying suspect-cases regardless of patient or professional preferences  Limitations---see above (item 18). |
| Implications | 20 | D;V | Discuss the potential clinical use of the model and implications for future research. | 26−31 | This study developed two prediction scores, which included fatigue, difficulty in swallowing, confusion-disorientation, red eyes, gingival bleeding, hematemesis, hemoptysis, and any history of contact with an EVD patient as a diagnostic predictor, some of which and their accuracy performance was consistent with those previously developed [21,22,36]. Although the methods used to develop prediction scores were different from ours, both models demonstrated better performance in discriminating EVD cases from others. Additionally, this discriminative performance remained the same, thus robust, in the two stratified sets of sample, e.g. for the set who fitted or did not fit the WHO case definition for the EVD suspects. The highest sum sensitivity-specificity is at -1 point and -1 point, respectively, for the clinical prediction score and the extended one; the models are associated with sensitivity of 85% and 42.3% and specificity of 78.8% and 81.4%. This means, for our prediction scores, the presence of a positive numeric score will be a useful argument for more investigations and/or the isolation of a suspect case. However, instead of choosing a sole cut-point, it is worth to divide it into three or four categories based on the numerical score, describing different probability levels of being useful in clinical practice. For instance, for these two models, one can consider a score of prediction equal or less than -2 as having a very low probability of the disease (i.e., less than 20%) and a score of prediction greater than +2 as having very high probability of the disease and act accordingly. With intermediate probabilities, e.g., between these two levels of predictive disease probabilities, one should consider the prediction score as not conclusive and call for additional clinical evaluation. In this sense, using these models can allow the decision to be made on at least 40.7 percent (4141 non-EVD and 114 EVD cases) and 79.8% (7965 non-EVD and 360 EVD cases), respectively, for the clinical prediction score and the extended score.  Therefore, using these prediction scores can reduce the number of unnecessary isolation and/or additional procedures to be carried out for suspect-cases. The two predicting scores are associated with a very low probability for EVD, and they avoid any delay in the isolation and case management for subjects with very high probabilities of disease in the isolation wards. Another way of using this score is to set up a threshold based on the greater sum sensitivity-specificity and then apply it to all suspect-cases. Those with scores above the threshold can be isolated whereas subjects with a score under the threshold would not. However, the choice of the operational cut-off score should not only depend only on sensitivity and specificity but also on how professionals or patients weigh the errors of classification (false-positive and negative). Notably, the availability of human resources and health infrastructures and the context of use must be taken into account while managing the two prediction scores. For instance, in a context with adequate infrastructure, available effective drugs, a "disease-averse" context ("worried about the disease"), it is worth choosing a sensitive operational cut-off to catch a maximum number of EVD cases. A sensitive threshold can reduce the transmission in the community, and can be held to separate suspect-cases within the isolation wards to reduce cross-contamination between true-positive and false-positive cases. Conversely, in a context with poor health infrastructure and low density of the population, an "isolation-averse" context (e.g., "worried about the isolation" context), choosing a high specific cut-point (avoiding the FP) can reduce the risk of nosocomial infection among the patients with low-risk of contamination at the community level. With caution, prediction scores could be useful tools in the settings with limited-resources. The prediction scores do not intend to substitute the rational clinical or epidemiological judgments but to provide additional information, which can help healthcare workers to decide whether to isolate an EVD suspect case.  Clinical usefulness of the developed models  Furthermore, we assessed the clinical usefulness of the two predictions scoring models developed by AUROC and the net benefit of using them to isolate EVD suspect-cases. We noted that both diagnostic prediction scores provided significantly higher AUROC and NB values relative to using clinically reasonable strategies (e.g., isolating all or none of the suspects). The AUROC is a summary measure of discrimination between individuals who experienced the disease and those who did not. Though it is subjective to determine the good value of the AUROC for assessing the disease risk, the AUROC values for the two scores developed are considered excellent, according to D’Agostino’s AUROC classification [37]. In addition, the two final scoring algorithms are quite robust for the selected variables. However, accuracy measures only tell us about the models or tests' discriminative performance; they do not consider the clinical consequences of using these clinical prediction models to inform on their clinical usefulness in making decisions [29]. While addressing this issue, the study has also shown that incorporating prediction models into the decision-making to isolate suspect-cases would improve clinical results or outcomes linked to the use of WHO case definition for suspect-cases while awaiting the confirmation results. Their use avoids many unnecessary isolations and only few true EVD cases could miss the isolation. Our findings demonstrate the utility of these models while identifying suspect-cases regardless of patient or professional preferences. The application of the DCA corresponds to the situation in which patients EVD suspect-cases do not have a definite diagnosis yet. The healthcare workers or clinical team must decide on whether the suspect-cases should be isolated or not, while the isolation exposes them to the cross-contamination among isolated people. This decision depends on the posterior probability of the disease for a suspect. Practically, the decision is straightforward if the suspected-case has a very low or very high probability, e.g., less than 20% or greater than 80%, to have the disease. For intermediate probabilities, the decision is more complex and will require a consensus. The decision should consider the EVD’s risk of spread-out (i.e., in communities or healthcare stings), disease severity and the context in which the decision is taken, including the availability of the resources, and should be also discussed on the basis of the perspective of the decision, which can be patient-centered, healthcare-centered, or societal-centered. Again, the DCAs represent rapid tools in the decision-making of healthcare workers during the surveillance and at the screening at point of-entry since they do not require much additional data in the analysis. Nonetheless, they do not substitute traditional decision analysis, e.g., cost-effectiveness analysis, when their use is to inform and direct a policy choice. |
| **Other information** | | | | |  |
| Supplementary information | 21 | D;V | Provide information about the availability of supplementary resources, such as study protocol, Web calculator, and data sets. | 34 | **Data Availability Statements:**  Due to ethical restrictions related to patient confidentiality, all relevant data is available upon request by emailing Dr. Antoine Tshomba ([antotshomba@yahoo.fr](mailto:antotshomba@yahoo.fr)) |
| Funding | 22 | D;V | Give the source of funding and the role of the funders for the present study. | 33 | This study has received partial support from NIH (Grant NIH FIC/ R01EY031894). The funder had no role in the design of the study; in the collection, analyses, or interpretation of data; in the writing of the manuscript, or in the decision to publish the results |

*Items relevant only to the development of a prediction model are denoted by D, items relating solely to a validation of a prediction model are denoted by V, and items relating to both are denoted D;V. We recommend using the TRIPOD Checklist in conjunction with the TRIPOD Explanation and Elaboration document.
